# Dynamics of usage of menstrual hygiene and unhygienic methods among young women in India: a spatial analysis

**DOI:** 10.1186/s12905-023-02710-8

**Published:** 2023-11-06

**Authors:** Trupti Meher, Harihar Sahoo

**Affiliations:** International Institute for Population Sciences (IIPS), Deonar, Mumbai India 400088

**Keywords:** Menstrual hygiene practices, Hygienic methods, Unhygienic methods, Young women, India

## Abstract

**Background:**

Menstruation, especially the menstrual cycle, is a vital sign for female adolescent health and maintaining menstrual hygiene is of utmost importance for menstruating girls and women. However, menstrual hygiene and management are issues that have not received adequate attention. Therefore, the present study aimed to explore spatial patterns of menstrual hygiene practices in India and to identify their socioeconomic and demographic determinants among women aged 15–24 years.

**Methods:**

The study utilized data from the fifth round of the National Family Health Survey (NFHS-5) conducted during 2019–21 in India. The analysis was limited to 241,180 women aged 15–24 years. The statistical methods range from multinomial logistic regression, spatial autocorrelation in terms of Moran’s I statistics, to spatial regression in order to understand the spatial dependence and clustering in different methods of menstrual practices across the districts of India.

**Results:**

Almost half of the respondents (49.8 percent) reported using hygienic methods of bloodstain protection, while 22.7 percent still relied on unhygienic methods and 27.5 percent reported using both hygienic and unhygienic methods during their menstruation. Factors like age, place of residence, caste, religion, education, wealth index and toilet facility were found to be significantly associated with the use of unhygienic and both methods. It was also observed that the percentage of women practicing hygienic methods was predominantly higher in the Southern region. On the other hand, states like Madhya Pradesh and Bihar appeared to be hotspots for unhygienic menstrual practices. The univariate Moran’s *I* value for unhygienic and both methods were 0.722 and 0.596, respectively, depicting high spatial autocorrelation across districts in India. In spatial regression, rural residence, illiteracy, poverty, and no toilet facility were found to be statistically significant predictors of the use of unhygienic method and both methods.

**Conclusion:**

Young women should be educated about the importance of menstrual hygiene practices and the physiological consequences of unhygienic practices. Furthermore, interventions should target socio-economically disadvantaged women to increase the use of sanitary napkins.

## Introduction

Menstruation, especially the menstrual cycle, is a vital sign for female adolescent health [1]. It is a completely natural and biological phenomenon among mature women who experience the shedding of blood for 1–7 days every month until menopause. Though menstruation is a physiological process, it is generally considered unclean, dirty, or impure and is shrouded in misconceptions, taboos, and stigma in many societies [2–4]. Considering menstruation to be an uncomfortable and embarrassing topic, it is often not discussed openly. Due to a lack of open conversation with mothers or other women, young girls in both rural and urban regions often grow up with little awareness about menstruation [5, 6]. Even today, in many families, menstruation is a secret between mother and daughter. In general, when girls have their menstruation for the first time, they have very little knowledge of the physiological processes linked with sexual development, biological facts, and menstrual management methods [7, 8].

Maintaining menstrual hygiene is of utmost importance for menstruating girls and women. Menstrual hygiene is defined as the principle of maintaining the cleanliness of the body during menstruation. It necessitates simple amenities such as hygienic absorbent material, water, soap, and toilet facilities with privacy [9]. An essential component of managing menstrual hygiene is the selection and effective usage of menstrual absorbents. According to a review by Chandra-Mouli et al. [10], the degree of menstrual monitoring of adults may contribute to variances in core menstrual hygiene management (MHM) behaviours such as absorbent usage, frequency of change, and daily bathing routines, etc. According to the World Health Organisation (WHO), around 1.7 billion people globally lack access to basic sanitation [11]. In addition, owing to high cost and lack of knowledge, women often use old clothes and other unhygienic material as menstrual absorbents. Poor personal hygiene and unhygienic menstrual practices often lead to serious and long-term health issues like gastrointestinal, genital, and perineal infections, recurrent reproductive tract infections (RTIs) and even cervical cancer [12–14]. In addition, menstruating girls frequently face unnecessary restrictions in their daily lives as a result of inadequate and inaccurate knowledge about menstruation, which can lead to a range of psychological problems [15].

Menstrual hygiene practices are influenced by a variety of factors, including women’s knowledge and understanding of menstruation, the availability of appropriate facilities and the socio-cultural environment [16–18]. Studies have shown that menstrual behaviours and understanding are influenced by several socioeconomic and demographic factors as well [19, 20]. According to a study by Kuhlmann et al. (2017) [21], in several low- and middle-income countries, a large chunk of the female population practices unhygienic methods during menstruation, with a majority of them coming from rural areas. Previous studies on menstrual practices among adolescent girls in India have reported the utilization of sanitary pads ranging from 20 to 35 percent in different regions of the country [20, 22, 23]. Moreover, a recent study in India showed that nearly 62 percent of young women use old clothes to prevent bloodstains during menstruation [19]. Unhygienic menstrual practices are more prevalent among rural and poor women [12]. Among young women, unhygienic menstruation practises are exacerbated by insufficient information passed down from their mothers, who are themselves ignorant of reproductive health and hygiene due to illiteracy and low socioeconomic status [24]. Moreover, the lack of access to sanitary products and adequate sanitary facilities in poorly resourced nations is a significant barrier for menstruating women.

Menstrual hygiene and management are issues that have not received adequate attention. According to the Census of India (2011), women aged 15 to 24 account for nearly 19 percent of the total female population in the country. Therefore, it is important to pay careful attention to the monthly menstrual needs of such an enormous population. Considering the above scenario, the present study was conducted among young Indian women aged 15–24 years. Although numerous attempts have been made to study menstrual practices in India, most of them have focused on knowledge, awareness and health issues, with only a few research based on nationally representative data. Nevertheless, there is research focusing on the spatial patterns of menstrual hygiene practices in the country; however, studies demonstrating geospatial patterns and determinants of the exclusive use of hygienic, unhygienic and the use of both methods are rare. In addition, an in-depth understanding of the intricacy of the issue as well as the associated factors that may possibly impact the menstrual practices of Indian women is required for efficient menstrual hygiene management for women. Therefore, the present study aimed to explore spatial patterns of menstrual hygiene and unhygienic methods in India and to identify their socioeconomic and demographic determinants. The findings of this study might aid in planning, formulating policies, and creating effective intervention strategies.

## Methods

### Data source

The data for this study was drawn from the fifth round of the National Family Health Survey (NFHS-5), which was conducted during 2019–21 in India. The International Institute for Population Sciences (IIPS) served as the nodal agency to carry out the survey under the stewardship of the Ministry of Health and Family Welfare (MoHFW), Government of India, with technical support from ICF International. The NFHS is a large-scale, multi-round survey conducted in a nationally representative sample of households. The fundamental objective of this survey was to obtain national and state-level estimates on fertility, family planning, reproductive health, nutrition, maternal and child health, women’s autonomy, domestic violence, etc. Each successive round of the NFHS has two specific goals. One is to provide essential data on health and family welfare needed by the Ministry of Health and Family Welfare and other agencies for policy and programme purposes, and the other is to provide information on important emerging health and family welfare issues. IIPS, being the nodal agency, was responsible for obtaining ethical approval for conducting and disseminating the survey data.

The NFHS-5 used a stratified two-stage sampling method. Primary sample units (PSUs) were chosen in the first step utilizing population proportion to size sampling (PPS). Villages were regarded as PSUs in rural regions, whereas Census Enumeration Blocks (CEBs) were PSUs in urban areas. The second stage involved selecting 22 households per cluster using equal probability systematic sampling. A total of 664,972 households were chosen for the sample, with 636,699 households successfully interviewed and a response rate of 98 percent. Furthermore, 747,176 eligible females aged 15–49 years and 111,179 eligible males aged 15–54 years were selected for individual interviews. However, interviews were conducted with 724,115 women, with a response rate of 97 percent, and 101,839 males, with a response rate of 92 percent. The current study's analysis was confined to young women aged 15–24 years, with a total sample size of 241,180 individuals.

### Outcome variable

The outcome variable for this study was menstrual hygiene practices. Women in the age group of 15–24 were asked a direct question on their menstrual practices i.e., “Women use different methods of protection during their menstrual period to prevent bloodstains from becoming evident. “What do you use for protection, if anything?” This information was used to carry out the analysis. However, the outcome variable was classified into three categories:

- Exclusive use of hygienic methods (sanitary napkins, locally prepared napkins, tampons, menstrual cups)
- Exclusive use of unhygienic methods (clothes, nothing, others)
- Combination of both hygienic and unhygienic methods

### Predictor variables

- *Age***:** Respondents were asked about their current age at the time of the survey. In this study, the age of respondents was classified into two groups: “15–19 years” and “20–24 years”.
- *Marital status***:** When respondents were asked about their current marital status, the responses were like “Never in union” (includes married but gauna not performed), “Married”, “Living with partner”, “Windowed”, “Divorced”, “Separated”. However, in this study, the variable was categorized into three groups: “Never in union”, “Currently married/Living with partner” and “Widowed/Divorced/Separated”.
- *Place of residence***:** It was categorized into two groups; “Urban” and “Rural”.
- *Region***:** The “state” variable was categorized into six groups in order to form region variable. These six groups were: “North”, “Central”, “Northeast”, “East”, “West”, “South”.
- *Caste***:** This variable was recoded into three categories, ‘Scheduled Caste/Scheduled Tribe’ (SC/ST), ‘Other Backward Classes’ (OBC), and ‘Others’.
- *Religion***:** Religion was recoded as “Hindu”, “Muslim”, “Christian” and “Others”.
- *Education***:** This variable describes the educational level of the respondents. It was recoded as: “No education”, “Primary”, “Secondary” and “Higher”.
- *Wealth index***:** This variable represents the economic status of the household of the respondents. Households were given scores based on the number and kind of consumer goods they own such as television, bicycle, car etc., as well as residential characteristics such as water supply, bathroom facilities, and flooring materials. These scores were computed using principal component analysis. The national wealth quintiles were determined by assigning a score to each household member, rating each individual in the household population based on their score, and then dividing the distribution into five equal groups, each with 20 percent of the population. This variable was recoded as: “Poorest”, “Poorer”, “Middle”, “Richer” and “Richest”.
- *Toilet facility***:** It was categorized into three groups: “Improved toilet facility”, “Unimproved toilet facility” and “No facility/open defecation”. Improved toilet facility includes toilet of the following types: flush/pour flush toilets to piped sewer systems, septic tanks, pit latrines, or an unknown destination; ventilated improved pit (VIP)/biogas latrines; pit latrines with slabs; and twin pit/composting toilets. Unimproved toilet facility includes flush/pour flush not to sewer/septic tank/pit latrine, pit latrine without slab/open pit, dry toilet and other.
- *Mass media exposure***:** Respondents were asked how often they read newspapers or magazines, listen to the radio, watch television, go to the cinema hall and use internet. Those who responded almost ‘every day’ and ‘at least once a week’ were considered to be regularly exposed to that form of media. The final version of mass media exposure variable was generated by adding the above-mentioned variables and was recorded as ‘no’ for zero exposure to mass media, ‘low’ for regular exposure to one or two mass media and ‘high’ for being regularly exposed to more than two mass media.

### Statistical analyses

The study started with a descriptive analysis of outcome and predictor variables. Bivariate analysis was performed to provide the percentage distribution of menstrual hygiene practices by the categories of predictor variables. Chi-square tests were performed to determine significant associations of explanatory variables with the outcome variable at p < 0.001. As the outcome variable had three categories (exclusive use of hygienic method, exclusive use of unhygienic method and both methods), multinomial logistic regression was used to find the determinants of use of ‘unhygienic’ and ‘both’ methods. Exclusive use of hygienic method was taken as the reference category in the regression model.

Additionally, spatial analysis of menstrual hygiene practices was performed using the software GeoDa. Univariate and bivariate Moran’s *I* statistics along with bivariate Local Indicators of Spatial Association (LISA) Cluster and Significance maps for women practicing unhygienic and both methods were estimated.

Moran’s *I* is the Pearson coefficient measure of spatial autocorrelation. It tells us whether a phenomenon is clustered spatially or not. The formula to compute the Moran’s I statistic is as follows:

$$I=\frac{N}{W}\frac{{\Sigma }_{i}{\Sigma }_{j}{w}_{ij}\left({x}_{i}-\overline{x }\right)\left({x}_{i}-\overline{x }\right)}{{\Sigma }_{i}{\left({x}_{i}-\overline{x }\right)}^{2}}$$

where, x is the variable of interest; $\overline{x }$ is the mean of x; w_**ij**_ is a matrix of spatial weights with zero on the diagonal (i.e., w_**ij**_ = 0); N is the number of spatial units indexed by i and j; and W is the sum of all w_**ij.**_

A Moran’s I value ranges from -1 (indicating perfect dispersion) to + 1 (perfect correlation), where positive values indicate the spatial clustering of similar values and negative values indicate the clustering of dissimilar values. A zero value indicates a random spatial pattern with no spatial autocorrelation.

Bivariate LISA measures the local correlation between the independent and dependent variables geographically. It indicates whether the spatial distributions of the dependent variable and the independent variables are interrelated. The bivariate LISA involves the cross product of the standardized values of one variable at location i with those of the average neighbouring values of another variable.

A set of regression models was used to estimate significant correlates of the use of unhygienic method and the use of both methods and to identify the best fit model for analyzing them spatially. The Ordinary Least Square (OLS) model was run to examine the association between the dependent and independent variables.

Spatial lag model (SLM) and spatial error model (SEM) were also used after finding that Moran’s *I* for unhygienic and both practices were statistically significant. Spatial lag model suggests that the units are spatially dependent to each other and lagging to each in the nearby spatial locations.

SEM evaluates clustering of an outcome variable that is not explained by the independent variables. Here, spatial clustering is explained with reference to the clustering of the error terms.

In order to choose the best fit model, Akaike Criterion Values (AIC) were examined and compared between spatial lag and error models.

## Results

**Fig. 1:**
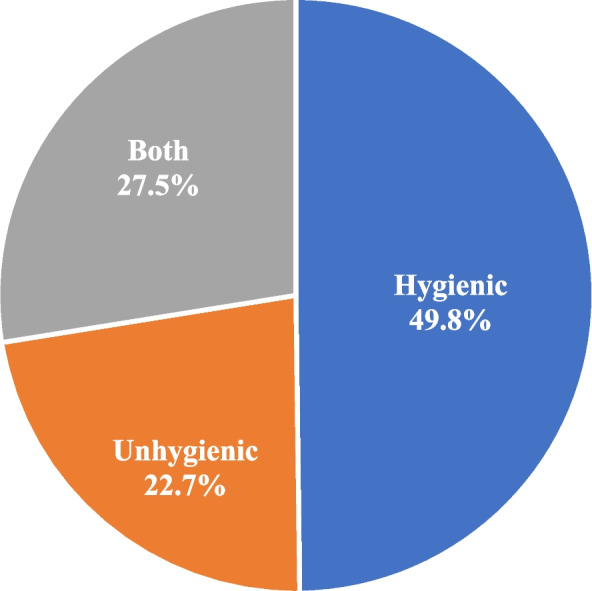
Percentage distribution of menstrual hygiene practices in India, NFHS-5

Figure 1 demonstrates the level of menstrual hygiene practices among women in India. Overall, almost half of the respondents (49.8 percent) used hygienic methods of bloodstain protection during menstruation. However, 27.5 percent reported using both hygienic and unhygienic methods.

### Differentials in menstrual hygiene practices

Table 1 shows the percentages of women practicing hygienic, unhygienic and both methods by background characteristics. In terms of marital status, the practice of hygienic methods was more prevalent among never married women (53 percent). However, the practice of unhygienic methods (34.8 percent) and both methods (29.2 percent) were quite common among widowed/divorced/separated women. The use of hygienic methods was higher among urban resident (68.1 percent) than their rural counterparts (42.2 percent). Considering the caste of respondents, the use of hygienic methods was observed to be the lowest among ST women (38.6 percent) as compared to all other caste groups. Moreover, it was observed that about 69.2 percent of women with higher educational attainment use hygienic methods as compared to 18.7 percent of illiterate women. Likewise, in the case of wealth index, the prevalence of hygienic methods increased and the prevalence of unhygienic methods decreased with the increase in wealth quintiles. Women living in households with improved toilet facilities showed a higher prevalence of hygienic methods (54.7 percent), while women using open defecation were more likely to opt for unhygienic methods (40 percent). Moreover, the practice of hygienic methods was considerably higher among women who had high exposure to mass media (65.3 percent) as compared to women who had no exposure to mass media (43.7 percent).

**Table 1:** Percentage distribution of menstrual hygiene practices by background characteristics, NFHS-5

Background characteristics	Menstrual Hygiene practices			*p* values	*N*
	**Hygienic (%)**	**Unhygienic (%)**	**Both (%)**		
**Age group**				*p* < 0.001	
15–19 years	50.0	22.5	27.6		1,22,480
20–24 years	49.6	22.9	27.5		1,18,700
**Marital status**				*p* < 0.001	
Never in union	53.0	19.4	27.6		1,58,224
Currently married/Living with partner	44.4	28.2	27.4		81,557
Widowed/Divorced/Separated	36.0	34.8	29.2		1,399
**Place of Residence**				*p* < 0.001	
Urban	68.1	10.6	21.3		54,561
Rural	42.2	27.7	30.1		1,86,619
**Region**				*p* < 0.001	
North	63.6	12.2	24.2		48,694
Central	31.4	30.4	38.2		64,401
East	44.1	28.6	27.4		42,998
Northeast	34.1	30.9	35.0		31,970
West	63.8	21.3	14.9		22,023
South	70.3	9.2	20.4		31,094
**Caste**				*p* < 0.001	
SC	48.4	23.6	28.0		49,136
ST	38.6	34.6	26.7		44,392
OBC	48.3	22.6	29.1		93,969
Others	58.3	17.2	24.5		53,683
**Religion**				*p* < 0.001	
Hindu	50.2	22.7	27.2		1,81,475
Muslim	43.1	25.6	31.3		33,773
Christian	63.0	14.5	22.5		15,802
Others	67.9	11.6	20.5		10,130
**Education**				*p* < 0.001	
No education	18.7	56.7	24.6		16,010
Primary	25.1	46.7	28.2		15,082
Secondary	49.6	21.5	28.9		1,68,132
Higher	69.2	7.4	23.5		41,956
**Wealth Index**				*p* < 0.001	
Poorest	24.7	46.7	28.6		52,954
Poorer	37.5	29.2	33.3		57,319
Middle	51.9	18.2	29.9		51,275
Richer	63.2	11.1	25.7		44,733
Richest	76.9	5.0	18.1		34,899
**Toilet facility**				*p* < 0.001	
Improved toilet facility	54.7	18.0	27.4		1,79,822
Unimproved toilet facility	43.4	27.8	28.8		9,367
No facility/open defecation	32.2	40.0	27.8		42,893
**Mass media exposure**				*p* < 0.001	
No	43.7	27.4	28.8		1,76,405
Low	64.8	11.1	24.1		57,340
High	65.3	9.2	25.5		7,435

*SC* Schedule Caste, *ST* Schedule Tribe, *OBC* Other Backward Classes

### Factors associated with unhygienic and both methods of menstrual practices

Table 2 presents the results of the multinomial logistic regression analysis which was performed in order to identify the factors associated with unhygienic and both methods of menstrual practices among women aged 15–24 in India. As per the results, women aged 20–24 were significantly more likely to practice unhygienic (RRR = 1.189; 95% CI: 1.155–1.224) and both methods (RRR = 1.202; 95% CI: 1.173–1.233) for preventing menstrual bloodstains as compared to women who belonged to the age group of 15–19 years. Moreover, rural women were relatively more likely to opt for unhygienic (RRR = 1.637; 95% CI: 1.580–1.696) and both methods (RRR = 1.424; 95% CI: 1.386–1.463) of menstrual practice as compared to women from urban areas. Considering the region, women from the Central region of India were 3.145 times (95% CI: 3.029–3.265) and 2.417 times (95% CI: 2.345–2.492) more likely to practice unhygienic and both methods for the prevention of bloodstains compared to those from Northern India. Women from the upper caste were significantly less likely to use unhygienic methods (RRR = 0.920; 95% CI: 0.885–0.957) than women from the scheduled caste. In terms of religion, the relative risk for the use of unhygienic (RRR = 1.843; 95% CI: 1.778–1.910) and both methods (RRR = 1.613; 95% CI: 1.564–1.664) were significantly higher among Muslim women than Hindus. The regression analysis showed a significantly negative association between education and unhygienic menstrual practice. For instance, in comparison to uneducated women, women with higher education were relatively less likely to use unhygienic methods (RRR = 0.140; 95% CI: 0.132–0.149) for preventing menstrual bloodstains. Additionally, the analysis also showed a negative association of the wealth index with unhygienic and both methods of menstrual practice. In terms of toilet facilities, women from unimproved toilet households and those who use open defecation were significantly more likely to use unhygienic and both methods.

**Table 2:** Relative risk ratio (RRR) showing the effect of background characteristics on unhygienic and both method of MHP: results from multinomial logistic regression analysis, NFHS-5

Background characteristics	Unhygienic			Both		
	**RRR**	**95% CI**		**RRR**	**95% CI**	
		**Lower**	**Upper**		**Lower**	**Upper**
**Age group**						
15–19 years ®						
20–24 years	1.189***	1.155	1.224	1.202***	1.173	1.233
**Marital status**						
Never in union ®						
Currently married/Living with partner	1.185***	1.150	1.221	0.98	0.955	1.006
Widowed/Divorced/Separated	1.710***	1.484	1.971	1.174*	1.019	1.354
**Place of Residence**						
Urban ®						
Rural	1.637***	1.580	1.696	1.424***	1.386	1.463
**Region**						
North ®						
Central	3.145***	3.029	3.265	2.417***	2.345	2.492
East	1.226***	1.177	1.277	1.073***	1.037	1.111
Northeast	1.451***	1.382	1.524	1.334***	1.280	1.391
West	1.888***	1.803	1.978	0.735***	0.704	0.767
South	0.626***	0.594	0.659	0.639***	0.616	0.664
**Caste**						
SC ®						
ST	1.095***	1.053	1.139	0.923***	0.890	0.957
OBC	1.100***	1.065	1.135	1.068***	1.039	1.098
Others	0.920***	0.885	0.957	0.901***	0.872	0.931
**Religion**						
Hindu ®						
Muslim	1.843***	1.778	1.910	1.613***	1.564	1.664
Christian	0.799***	0.753	0.848	0.919**	0.873	0.967
Others	0.627***	0.585	0.671	0.805***	0.764	0.848
**Education**						
No education ®						
Primary	0.734***	0.690	0.781	0.945	0.884	1.010
Secondary	0.303***	0.289	0.317	0.679***	0.645	0.715
Higher	0.140***	0.132	0.149	0.510***	0.481	0.541
**Wealth Index**						
Poorest ®						
Poorer	0.535***	0.518	0.553	0.830***	0.804	0.858
Middle	0.319***	0.307	0.331	0.635***	0.613	0.657
Richer	0.201***	0.193	0.211	0.506***	0.486	0.526
Richest	0.102***	0.096	0.108	0.337***	0.322	0.352
**Toilet facility**						
Improved toilet facility ®						
Unimproved toilet facility	1.349***	1.275	1.427	1.079**	1.022	1.139
No facility/open defecation	1.300***	1.259	1.342	1.043**	1.011	1.076
**Mass media exposure**						
No ®						
Low	0.695***	0.673	0.717	0.928***	0.906	0.951
High	0.810***	0.747	0.878	1.084**	1.024	1.147

® Reference category; *, **, *** refers to < 0.05, < 0.01 and < 0.001 level of significance

*SC* Schedule Caste, *ST* Schedule Tribe, *OBC* Other Backward Classes

### Spatial pattern & clustering of menstrual hygiene practices

**Fig. 2:**
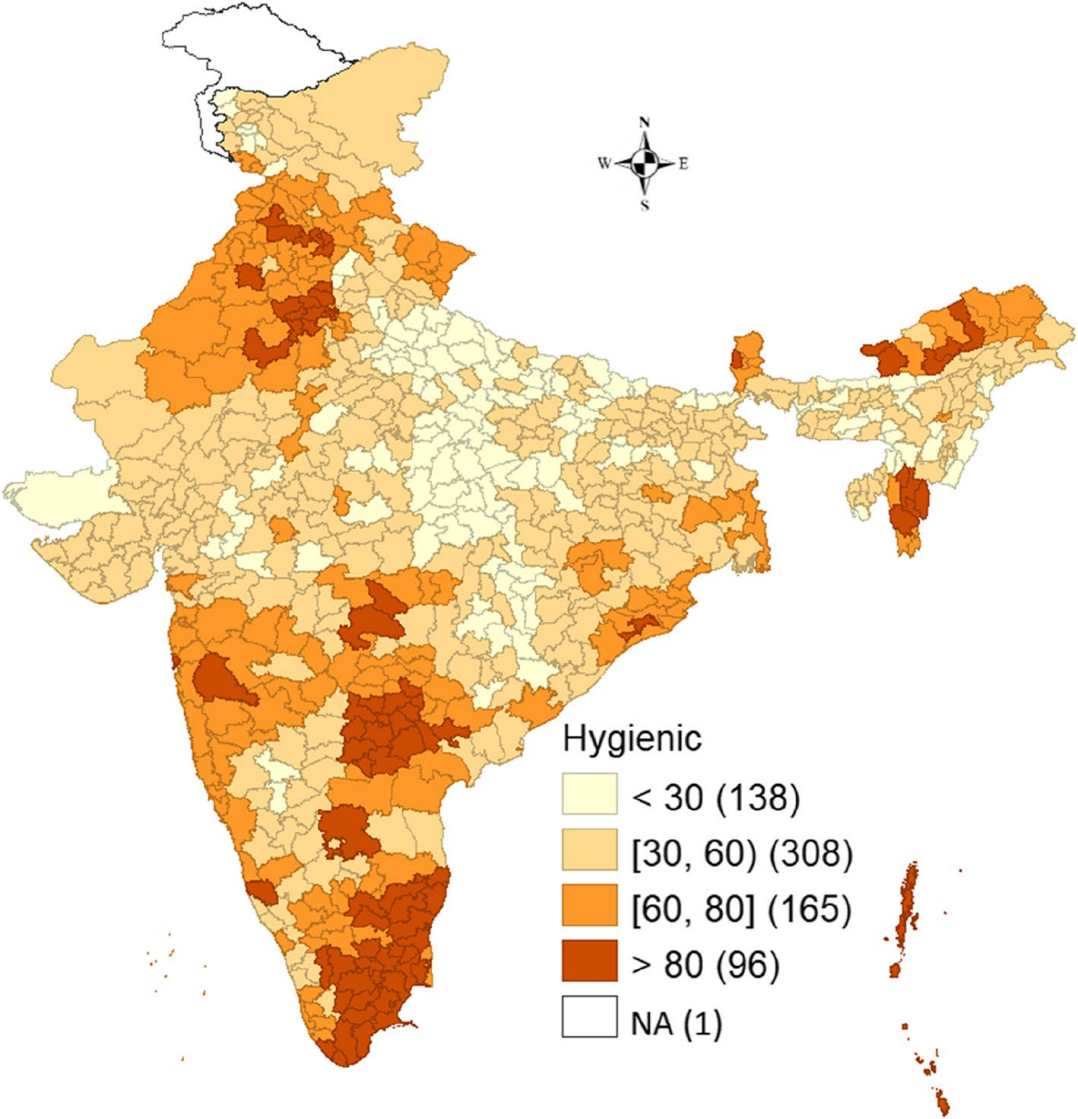
Spatial pattern of hygienic menstrual practices in districts of India, NFHS-5

Figure 2 shows the spatial pattern of hygienic menstrual practices in the districts of India. Ninety-six districts showed a high prevalence of hygienic menstrual practices (> 80 percent). Whereas 165 and 308 districts showed a prevalence between the ranges of 60–80 percent and 30–60 percent respectively. Lastly, 138 districts showed a low prevalence of hygienic menstrual practices (< 30 percent). It was also observed that the percentage of women practicing hygienic methods was predominantly greater in the districts of Tamil Nadu and Telangana. Additionally, most districts of Haryana and Mizoram and some districts of Arunachal Pradesh also showed a high prevalence of hygienic practices.

**Fig. 3:**
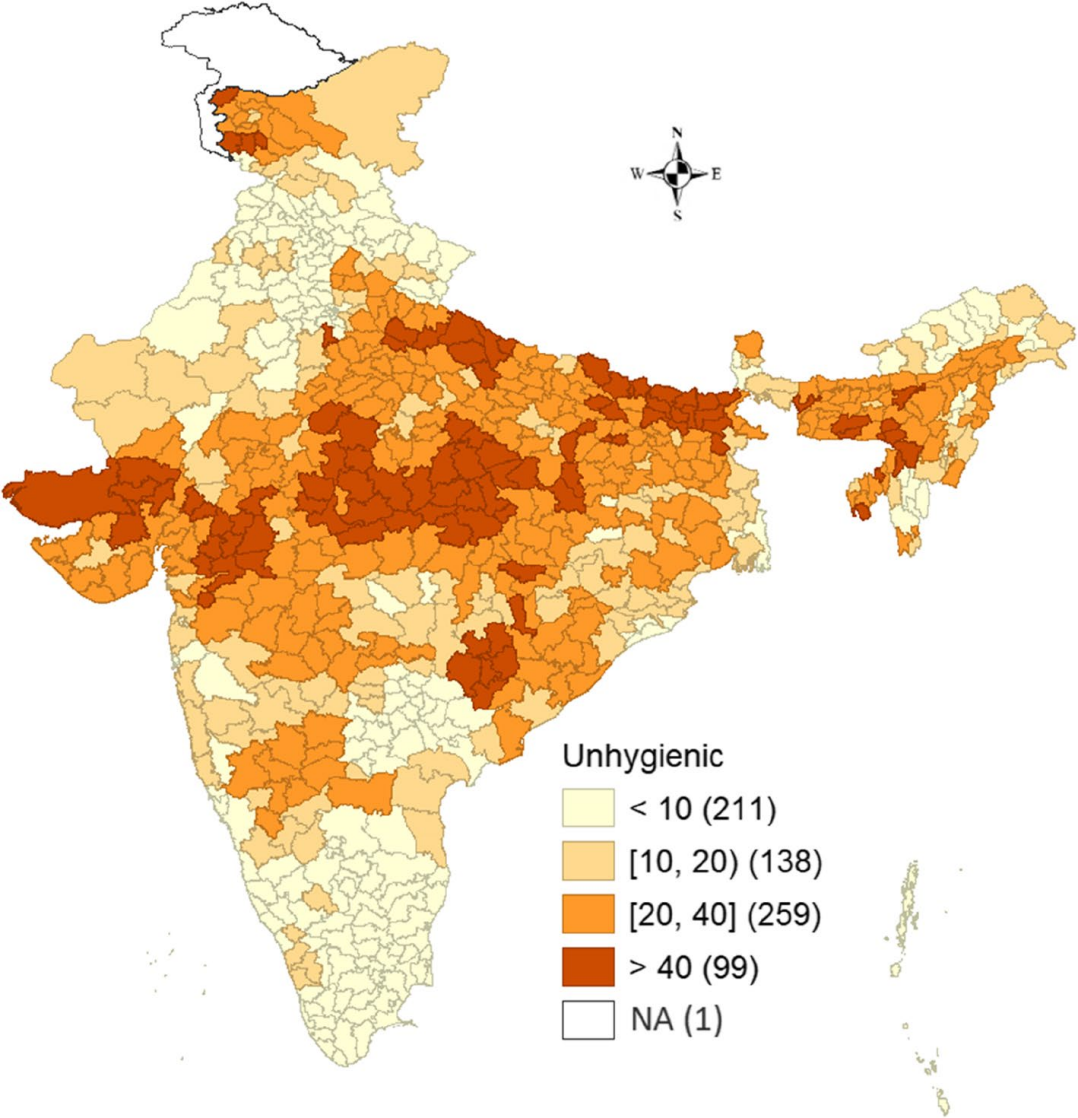
Spatial pattern of unhygienic menstrual practices in districts of India, NFHS-5

District level spatial pattern of unhygienic menstrual practices is presented in Fig. 3. The prevalence of unhygienic practices was considerably higher in the districts of Madhya Pradesh, Bihar and Gujarat. Moreover, a high prevalence of unhygienic practices was also recorded in some of the districts of Chhattisgarh and Uttar Pradesh. Furthermore, ninety-nine districts documented a high prevalence for unhygienic methods (> 40 percent), while 138 and 259 districts reported a prevalence estimate between 10–20 percent and 20–40 percent, respectively. Furthermore, 211 districts showed a low prevalence of less than 10 percent.

**Fig. 4:**
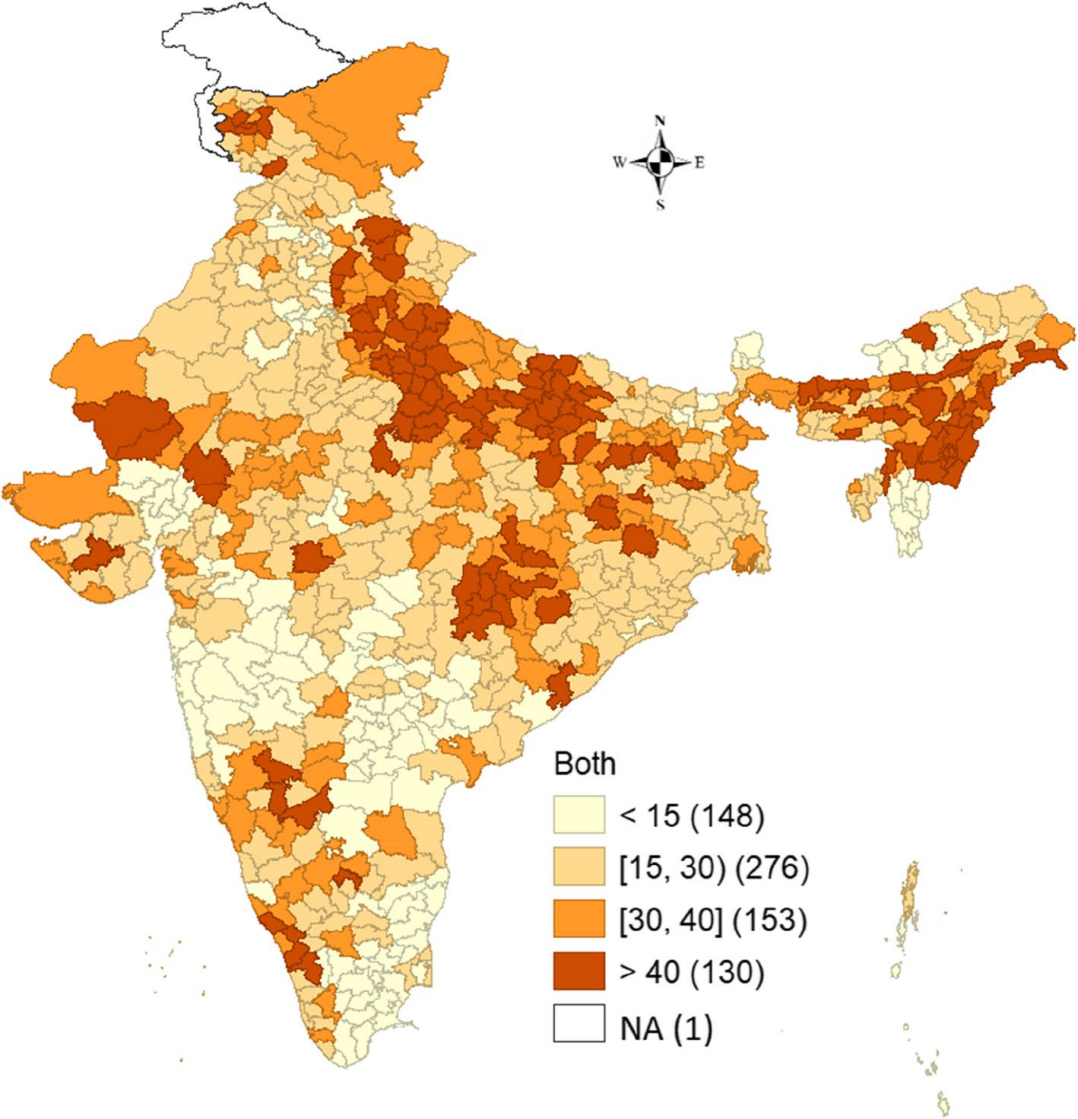
Spatial pattern of both hygienic and unhygienic menstrual practices in districts of India, NFHS-5

Figure 4 illustrates the spatial pattern of use of both hygienic and unhygienic methods of menstrual hygiene management. A majority of the districts in Uttar Pradesh and the Northeastern districts of Assam, Manipur and Nagaland and some districts of Chhattisgarh recorded high prevalence. Nearly 148 districts showed a low prevalence (< 15 percent) for the use of both methods, whereas a high prevalence (> 40 percent) was shown in 130 districts of the country. In addition, 276 districts had a prevalence of 15–30 percent and 153 districts showed a prevalence of 30–40 percent.

Table 3 presents the values of univariate and bivariate Moran’s *I* statistics. The univariate Moran’s *I* value for hygienic menstrual practice was 0.746, which depicts its high spatial autocorrelation across districts of India. The Moran’s *I* values were also high for the unhygienic method (0.722) and both methods (0.596). It was found that, among the independent variables, the spatial autocorrelation of the poorest wealth quintiles (0.741) and muslim (0.718) was the highest. It was also high for the SC/ST caste group (0.625), illiterate (0.583), households with no toilet facility (0.693) and zero mass media exposure (0.663). Lastly, the spatial autocorrelation for the 15–19 age group and rural area was 0.321 and 0.429, respectively.

**Table 3:** Moran’s *I* for dependent and independent variables

Variables	Univariate		Bivariate			
			**Unhygienic**		**Both**	
	**Moran's *I***	***p* value**	**Moran's *I***	***p* value**	**Moran's *I***	***p* value**
Hygienic methods	0.746	0.001	-	-	-	-
Unhygienic methods	0.722	0.001	-	-	-	-
Both methods	0.596	0.001	-	-	-	-
15–19 years	0.321	0.001	0.265	0.001	0.216	0.001
Rural	0.429	0.001	0.327	0.001	0.226	0.001
SC/ST	0.625	0.001	0.064	0.001	-0.015	0.183
Muslim	0.718	0.001	0.100	0.001	0.157	0.001
Illiterate	0.583	0.001	0.395	0.001	0.109	0.001
Poorest	0.741	0.001	0.540	0.001	0.268	0.001
Unimproved toilet	0.579	0.001	0.126	0.001	0.113	0.001
No toilet facility	0.693	0.001	0.371	0.001	0.029	0.047
No media exposure	0.663	0.001	0.420	0.001	0.239	0.001

### Bivariate LISA maps

**Fig. 5:**
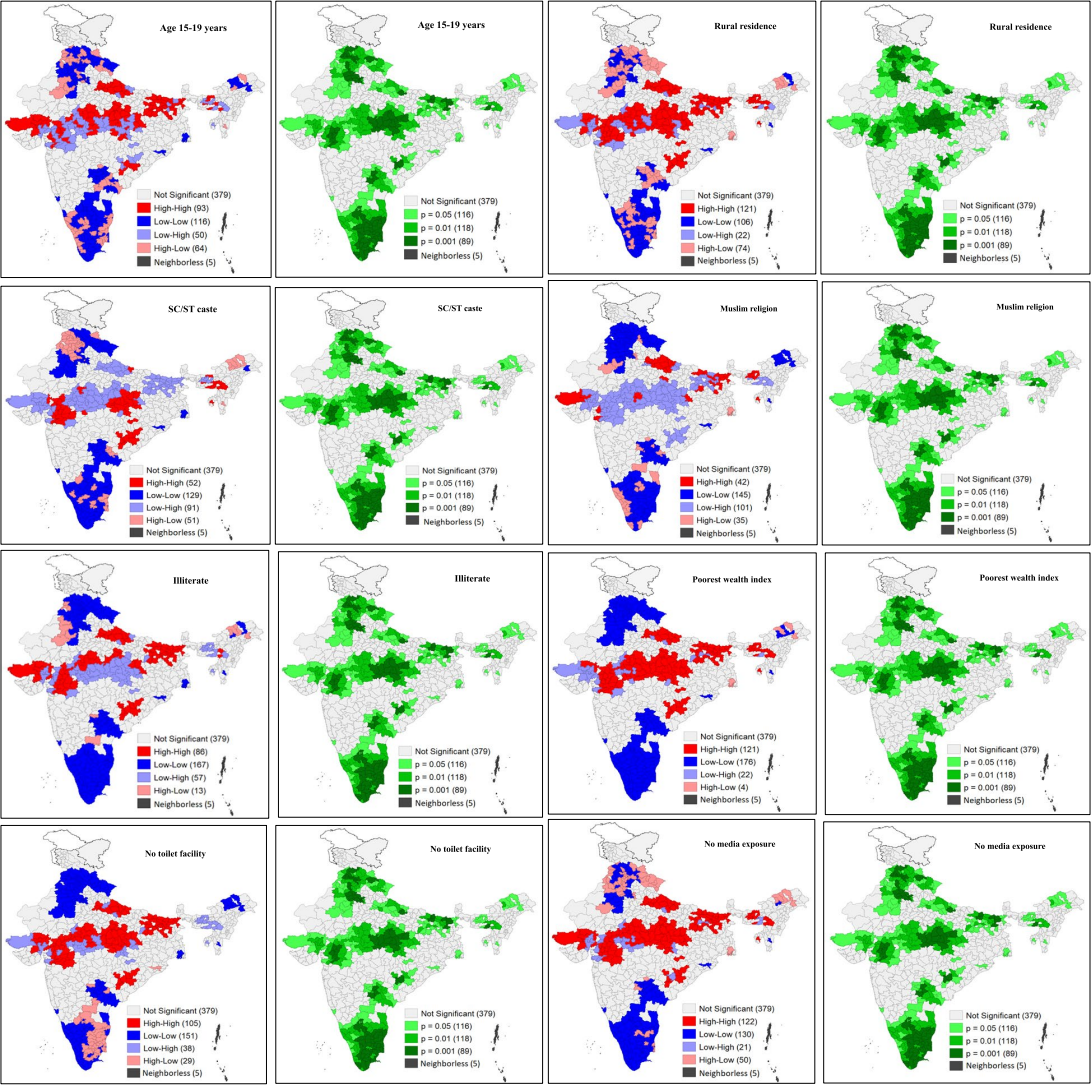
Bivariate LISA cluster and significance maps showing the spatial clustering of use of unhygienic methods and covariates

Figure 5 consists of bivariate LISA cluster and significance maps, which show the clustering of unhygienic methods of menstrual practice with its covariates across districts of India. Map 5.1 shows that 93 districts constituted the hotspot (high proportion of women aged 15–19 years and high proportion of unhygienic menstrual practice), whereas 116 districts constituted the cold spot (low proportion of women aged 15–19 years and low proportion of unhygienic menstrual practice). The hotspot districts belonged mainly to the states of Gujarat, Madhya Pradesh, Bihar and Uttar Pradesh. In Map 5.2, around 121 districts were the hotspots, with a high percentage of women living in rural areas and high usage of unhygienic methods. On the other hand, 106 districts were cold spots. Here, the hotspots were found to be located in the districts of Gujarat, Madhya Pradesh, Assam, Bihar and Uttar Pradesh. Similarly, Map 5.3 and Map 5.4 showed the clustering of unhygienic method of menstrual practices for the SC/ST caste group and the muslim religion. In addition, Map 5.5 showed high clustering (high percentage of illiterate women and high use of unhygienic methods) in 86 districts, whereas 167 districts showed low clustering. The hotspot districts mostly belonged to the states of Bihar, Uttar Pradesh, Gujarat and Madhya Pradesh. As per Map 5.6, 121 districts were hotspots with high percentage of poorest women and high usage of unhygienic methods. On the other hand, 176 districts were the cold spots. The districts pertaining to high clustering were mostly from Madhya Pradesh, Bihar and Uttar Pradesh and a few from Odisha, Chhattisgarh, Gujarat and the Northeastern states. Map 5.7 identified a total of 105 hotspots (high percentage of women living in households with no toilet facility and high usage of unhygienic methods during menstruation) in the states of Bihar, Uttar Pradesh, Madhya Pradesh and Gujarat, whereas 151 districts showed cold spots. Furthermore, Map 5.8 depicted 122 hotspots (high percentages of women with no mass media exposure and high usage of unhygienic methods) in the states of Gujarat, Madhya Pradesh, Bihar, Uttar Pradesh and a few Northeastern districts.

**Fig. 6:**
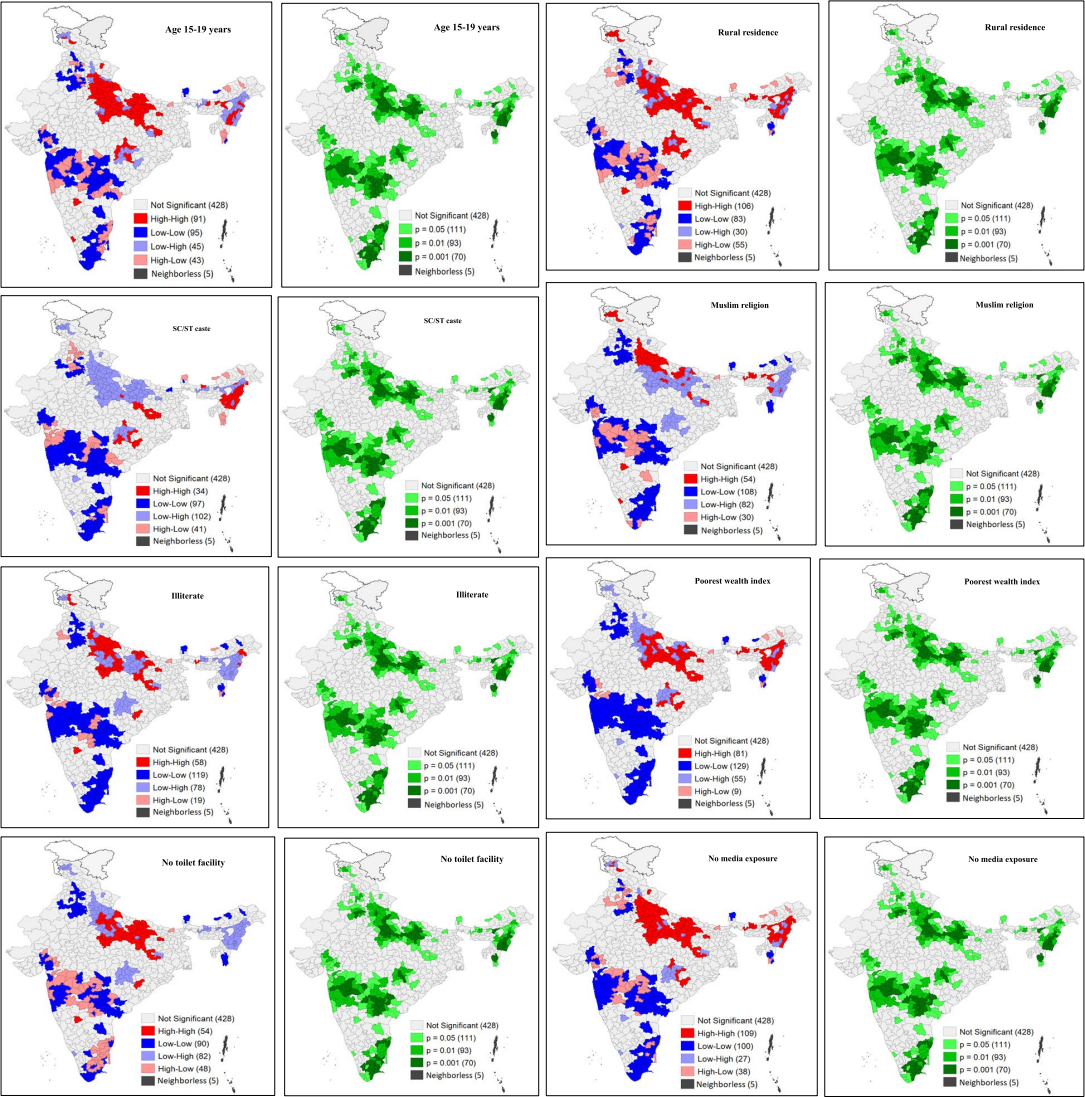
Bivariate LISA cluster and significance maps showing the spatial clustering of use of both methods and covariates

Figure 6 presents bivariate LISA cluster and significance maps depicting the clustering of both methods with the selected covariates. Map 6.1 showed that 91 districts constituted the hotspot (high proportion of women aged 15–19 years and high proportion of use of both methods), whereas 95 districts constituted the cold spot (low proportion of women aged 15–19 years and low proportion of use of both methods). The hotspot districts belonged mainly to the states of Uttar Pradesh and Chhattisgarh. A few Northeastern districts also came under hotspot districts. As per Map 6.2, around 106 districts were the hotspots, with a high percentage of women living in rural areas and high usage of both methods and 83 districts were the cold spots. Here, the major hotspots were found to be located in the districts of Uttar Pradesh and Chhattisgarh. Some Northeastern districts that belonged to the states of Assam, Manipur and Nagaland also showed high clustering. Maps 6.3 and 6.4 showed the clustering of unhygienic methods of menstrual practices for the SC/ST caste group and the muslim religion. Furthermore, Map 6.5 identified high clustering (high percentage of illiterate women and high use of both methods) in 58 districts, whereas 119 districts showed low clustering. The hotspot districts mostly belonged to the state of Uttar Pradesh. Whereas the cold spots were concentrated in the districts of Gujarat, Maharashtra, Tamil Nadu, Telangana and Andhra Pradesh. As per Map 6.6, around 81 districts were the hotspots with a high percentage of poorest women and high usage of both methods. On the other hand, 129 districts were the cold spots, with a low percentage of poorest women and low usage of both methods. Moreover, Map 6.7 showed a total of 54 hotspots (high percentage of women living in households with no toilet facility and high usage of both hygienic and unhygienic methods during menstruation) clustered in the districts of Uttar Pradesh, whereas 90 districts showed cold spots. In addition, Map 6.8 depicted 109 hotspots (high percentage of women having no mass media exposure and high usage of both methods) belonging to the districts of Uttar Pradesh, Assam, Nagaland and Manipur. However, 100 districts were identified as cold spots and primarily belonged to the states of Maharashtra, Telangana and Tamil Nadu.

### OLS, spatial lag and error models

Table 4 presents the results from OLS, SLM and SEM depicting the association between the predictor variables and unhygienic menstrual practices. In the OLS model, except for no media exposure, all the factors were found to be significantly associated with the use of unhygienic methods during menstruation. Factors like 15–19 age group, rural residence, SC/ST caste group, no educational attainment, poorest wealth quintile and no toilet facility in the household were significantly positively associated with the use of unhygienic methods during menstruation. However, muslim women showed a significantly negative association with unhygienic menstrual practices. Furthermore, in the cases of SLM and SEM, rural place of residence was positively associated with unhygienic menstrual practices. This was also the case for muslim, illiterate and poorest women as well as for women with no toilet facilities.

**Table 4:** OLS, Spatial Lag, and Error Model to assess the association between unhygienic method of menstrual practice and selected covariates, NFHS-5

Variables	OLS	S.E	SLM	S.E	SEM	S.E
15–19 years	0.325***	0.086	0.104	0.060	0.037	0.060
Rural	0.073***	0.021	0.073***	0.015	0.086***	0.017
SC/ST	0.050**	0.019	-0.024	0.013	-0.068***	0.018
Muslim	-0.05**	0.023	0.069***	0.016	0.094***	0.025
Illiterate	0.488***	0.073	0.328***	0.052	0.487***	0.065
Poorest	0.362***	0.030	0.160***	0.023	0.311***	0.030
No toilet facility	0.096**	0.033	0.053*	0.023	0.112***	0.033
No media exposure	0.064	0.335	-0.003	0.023	-0.007	0.032
**N**	707		707		707	
**Rho**			0.649***			
**λ**					0.813***	
**Akaike info criteria**	5194.55		4767.42		4679.50	
**R**^**2**^	0.604		0.805		0.841	

*, **, *** refers to < 0.05, < 0.01 and < 0.001 level of significance

Based on the Akaike Information Criterion (AIC) values, SEM was found to be the best fit model for all the predictors, as the AIC value was lowest in this model. The AIC value for SEM was 4679.50 and the error lag value (λ) was 0.813 (*p*- value < 0.001), while the value for R^2^ was 0.841.

Table 5 presents the results from OLS, SLM and SEM depicting the association between the predictors and the use of both hygienic and unhygienic methods during menstruation. In the OLS model, except illiterate and no media exposure, all other factors were found to be significantly associated with the use of both methods during menstruation. Factors like the 15–19 age group, rural residence, muslim religion, the poorest wealth quintile and no toilet facility in the household were significantly positively associated with the use of both methods. However, SC/ST women and women with no toilet facilities showed a significantly negative association with the usage of both methods. Moreover, in the cases of SLM and SEM, rural place of residence was positively associated with the use of both methods. This was also the case for women belonging to the poorest wealth quintile. On the other hand, women with no toilet facilities showed a significantly negative association with the use of both methods.

**Table 5:** OLS, Spatial Lag, and Error Model to assess the association between the use of both methods and selected covariates, NFHS-5

Variables	OLS	S.E	SLM	S.E	SEM	S.E
15–19 years	0.438***	0.103	0.162*	0.077	0.032	0.084
Rural	0.128***	0.026	0.095***	0.019	0.123***	0.023
SC/ST	-0.063**	0.023	-0.022	0.017	-0.003	0.024
Muslim	0.073**	0.028	0.037	0.021	0.06	0.033
Illiterate	-0.102	0.088	-0.045	0.065	-0.027	0.089
Poorest	0.213***	0.035	0.084**	0.027	0.136***	0.041
No toilet facility	-0.191***	0.040	-0.085**	0.030	-0.092*	0.045
No media exposure	0.045	0.040	-0.022	0.030	-0.088*	0.043
**N**	707		707		707	
**Rho**			0.674***			
**λ**					0.729***	
**Akaike info criteria**	5443.73		5110.850		5123.57	
**R**^**2**^	0.222		0.566		0.568	

*, **, *** refers to < 0.05, < 0.01 and < 0.001 level of significance

Based on the Akaike Information Criterion (AIC) values, the Spatial Lag Model was found to be the best fit model for all the predictors, as the AIC value was lowest in this model. The AIC value for SLM was 5110.85 and the rho value was 0.674 (*p*- value < 0.001), while the value for R^2^ was 0.566.

## Discussion

In the present study, the use of hygienic methods among young women aged 15–24 years was very low. Only half of the sampled population used hygienic methods during their menstruation. However, 23 percent of women reported the exclusive use of unhygienic menstrual absorbents such as old clothes, rags, etc. In addition, more than one-fourth of sampled women reported using a combination of both hygienic and unhygienic menstrual absorbents. Use of sanitary materials along with unhygienic materials can still pose several health risks, such as an increased risk of infections, skin irritation, odour, discomfort, etc. Previous research conducted in India also reported low levels of menstrual hygiene practices among Indian women [9, 25]. The present study has also identified several socio-economic and demographic factors associated with poor menstrual practices.

In accordance with previous research [26–28], the present study demonstrated that young women living in rural areas were more likely to practice poor menstrual hygiene than their urban counterparts. Moreover, the bivariate LISA map also demonstrated that rural areas in the Central region of the country have a high clustering of unhygienic menstrual practices. Rural women often hesitate to buy sanitary napkins from male shopkeepers because of the social stigma around it. This could be one of the possible reasons for unhygienic menstrual hygiene practices among rural women. Moreover, lack of awareness, taboos, and the unavailability and inaccessibility of hygienic menstrual absorbents are the major factors contributing to poor menstrual practices in rural India [29]. Furthermore, women belonging to scheduled tribes and caste groups were more likely to opt for unhygienic methods during their menstruation. This is in line with several previous research [24, 29]. SC/ST women from Madhya Pradesh and Chhattisgarh have reported a higher usage of unhygienic absorbents. Most of the tribal groups have historically been relegated to isolated and remote areas without proper access to markets or healthcare facilities. They are more likely to practice unsanitary measures of menstrual protection because of their isolation, restricted availability of disposable sanitary pads, and lack of awareness.

The present study demonstrated that an increase in educational and economic status was significantly associated with a decrease in the use of poor menstrual methods. Previous research has also demonstrated a similar kind of result [25, 28]. Again, LISA maps showed a high clustering of poor menstrual hygiene practices among women with no educational attainment and poorer economic backgrounds from Central India. One of the possible explanations behind the higher use of hygienic methods among women with a higher educational level as compared to illiterate women could be that they are better informed about the risks of unhygienic menstruation practices, have greater autonomy in making decisions, and are more financially independent. Additionally, studies have indicated that lower economic status is one of the key risk factors for poor menstrual practices [26, 30, 31]. The findings of the present study also revealed that women belonging to the higher wealth quintile are less likely to use poor menstrual hygiene practices as compared to those belonging to the lower wealth quintile. In our country, one of the biggest obstacles to menstrual hygiene management is the cost of sanitary products. Therefore, buying sanitary products might be expensive for women from low-income families. This study also revealed that poor menstrual hygiene practices are more common among women who live in households with unimproved toilet facilities and no toilet facilities. This corroborates the studies by Kathuria & Raj [32] and Singh & Anand [33].

From the spatial analysis, the following noteworthy findings emerge from this study. The results show that spatial patterns of menstrual hygiene practices are diverse across the districts of the country. The study further documented that factors like rural place of residence, illiteracy, lower wealth quintile and no toilet facilities were contributing to a significant increase in poor menstrual hygiene practices across the districts of India. The spatial dependency was high for rural residents, women belong to the age group of 15–19 years, poorest wealth quintile, no toilet facility, and no media exposure. Moreover, a majority of the districts in Tamil Nadu and Telangana showed high spatial clustering for the use of hygienic methods. This is in accordance with the study by Kathuria & Raj [32], which reported that the Southern region of India is performing better in menstrual hygiene management. The governments of these states have implemented schemes to promote menstrual hygiene and provide subsidized or free sanitary napkins to women, especially in rural areas. These initiatives have made sanitary napkins more accessible and affordable. In addition, there has been an improvement in the availability of sanitary napkins in these states. Increased distribution channels, including the establishment of vending machines and sanitary napkin banks, have made it easier for women to access these products. Furthermore, in the case of unhygienic menstrual practices, hotspots were found in most of the districts of Central India, including Madhya Pradesh, Chhattisgarh, Uttar Pradesh and districts from Gujarat and Bihar also showed high clustering. Ghose & Bose [19], in their study on menstrual hygiene management among Indian women, also found that women from Central India were more likely to practice unhygienic methods during their menstruation. India shows a wide range of geographical or regional variation in terms of socio-economic status, development, standard of living, and basic household amenities. This regional diversity influences menstrual hygiene practices among women across the country.

The study suffers from several limitations that should be taken into consideration while interpreting the results. Firstly, the cross‐sectional nature of the study does not allow the establishment of any causal relationship. Secondly, the self-reported nature of the data can be subject to recall or reporting biases. This may result in an underestimation or overestimation of the relationship between various background characteristics and menstrual hygiene practices. Nevertheless, one of the major strengths of this study is the wider relevance of its results since it is based on data from a large‐scale, nationally representative survey in India.

## Conclusion

In conclusion, the present study found poor menstrual hygiene practices among fifty percent of the sampled women. In addition, nearly one quarter of women reported the exclusive use of unhygienic methods. Moreover, the study has identified areas of the country with poor menstrual hygiene practices. This necessitates concentrating efforts to address unhygienic menstrual practices in these areas. Therefore, it is required to develop comprehensive menstrual health education programmes that address the biological, social, and emotional aspects of menstruation. Tailor the content to specific regions, languages, and cultural practices to ensure relevance and effectiveness. Furthermore, factors like age, place of residence, religion, educational level, wealth quintile and availability of toilet facilities were found to have a significant association with menstrual practices. In the past few years, government and non-governmental organizations have taken several initiatives for the improvement of menstrual hygiene management in India. However, these initiatives should be supported, expanded and broadened and more initiatives need to be taken through different projects or schemes, particularly among those representing socially, culturally, and economically marginalized communities. Frontline health professionals should be instructed to emphasize menstrual hygiene during routine home visits. Healthcare providers should be trained to deliver accurate and sensitive information about menstrual health and hygiene. It should be ensured that healthcare facilities have the necessary infrastructure and supplies to address menstrual hygiene needs effectively. Additionally, it is necessary to advocate for policies that prioritize menstrual health and hygiene, including provisions for affordable menstrual products, improved sanitation facilities, and comprehensive menstrual health education in schools.

## Data Availability

The data used in this research is publicly available on DHS website. Any individual can register and easily obtained data in electronic version from the following website https://dhsprogram.com/data/new-user-registration.cfm.

## Abbreviations

RTI: Reproductive Tract Infections
NFHS: National Family Health Survey
IIPS: International Institute for Population Sciences
MoHFW: Ministry of Health and Family Welfare
PSU: Primary Sampling Units
PPS: Population Proportion to Size
CEB: Census Enumeration Block
SC: Scheduled Caste
ST: Scheduled Tribe
OBC: Other Backward Classes
LISA: Local Indicators of Spatial Association
OLS: Ordinary Least Square
SLM: Spatial Lag Model
SEM: Spatial Error Model
AIC: Akaike Criterion Value
RRR: Relative Risk Ratio
CI: Class Interval
AIC: Akaike information criterion value
